# Comparison of the Differing Impacts of Lowered N-Acetylglucosaminyltransferase-Ia/b Activity on Motor and Sensory Function in Zebrafish

**DOI:** 10.3390/ijtm5030036

**Published:** 2025-08-18

**Authors:** M. Kristen Hall, Cody J. Hatchett, Haris A. Khan, Hannah Lewis, Ruth A. Schwalbe

**Affiliations:** Department of Biochemistry and Molecular Biology, Brody School of Medicine, East Carolina University, Greenville, NC 27834, USA

**Keywords:** N-glycans, oligomannose, N-acetylglucosaminyltransferase, GnT-I, zebrafish, anxiety, sensory function, motor activity

## Abstract

**Background/Objectives:**

Perturbation in terminal N-glycan processing is a feature of congenital disorders of glycosylation and neurological disorders. Since treatment options are limited, N-glycans are plausible therapeutic targets. Here, we investigated the consequences of substituting complex/hybrid with oligomannose types of N-glycans on nervous and musculature systems, employing *mgat1a* and *mgat1b* mutant zebrafish models.

**Methods:**

CRISPR Cas9 technology was employed to engineer the *mgat1a* zebrafish model. The N-glycan populations in Wt AB, *mgat1a*−/− and *mgat1b*−/− zebrafish were characterized via lectin blotting. Motor and sensory functions were measured by tail-coiling and touch-evoked response assays in embryos and larvae. Swimming locomotion and anxiety-like behavior were characterized in adult Wt AB, and mutant zebrafish using motility and novel tank dive assays.

**Results:**

The *mgat1a*−/− model had increased oligomannosylated proteins compared to Wt AB in embryos and dissected brain, spinal cord, skeletal muscle, heart, swim bladder, and skin from adults, supporting a global knockdown of GnT-I activity. Higher levels were also observed in *mgat1a*−/− relative to *mgat1b*−/−, except in the brain. Band patterns for oligomannosylated proteins were different between all three zebrafish lines. The *mgat1*−/− embryos and larvae had deficient motor and sensory functions which persisted into adulthood, with a higher deficiency in *mgat1b*−/−. Anxiety-like behavior was decreased and increased in adult *mgat1a*−/− and *mgat1b*−/−, respectively, compared to Wt AB.

**Conclusions:**

Taken together, this study revealed that aberrant terminal N-glycan processing impacts brain, spinal and muscle control, and hence will enhance our understanding of the vital role of complex/hybrid N-glycans in nervous system health.

## 1. Introduction

Glycosylation is a process involving complex co- and post-translation protein modifications via the addition of glycans. The three basic types of N-glycans (oligomannose, hybrid, and complex) all share a common pentasaccharide core and are processed sequentially [1]. The various types of N-glycans occur due to the addition of different branch points via the action of N-acetylglucosaminyltransferases (GnTs). These enzymes are encoded by the *MGAT* genes and are critical to the proper development of organisms at the cellular level. The conversion of oligomannosylated proteins into hybrid type is catalyzed via GnT-I, an enzyme encoded by the *MGAT1* gene. GnT-II, encoded by *MGAT2*, acts to further process hybrid- to complex-type N-glycans [1]. Since most proteins following the secretory pathway undergo N-glycosylation processing and this process can modify the structure and function of a protein, N-glycan processing is vital to the development and maintenance of a multicellular organism.

The magnitude of disruptions in N-glycosylation is highlighted in congenital disorders of glycosylation (CDG). Although CDG’s are a rare group of disorders, the number of identified CDGs are rising, and patients face a bleak prognosis as therapeutic options are quite limited, with dietary supplementation as the predominant management technique [2]. The impact of CDGs is multisystemic, with profound neurological complications [3,4]. Neurological symptoms associated with CDG include psychomotor retardation, cognitive disorders, ataxia, epileptic seizures, polyneuropathy, hypotonia, and stroke-like events [5,6]. Further, patients often experience depression and anxiety [7]. Like CDG’s, many other diseases have been associated with defective glycosylation, including cancer, neurodegenerative diseases, neurological disorders, and autoimmune diseases [5,8]. As such, additional research is necessary to further advance the field on the relationship between glycans and disease onset, progression, and treatment.

The diverse glycobiome of zebrafish (Danio rerio) has allowed for the creation of a platform to generate glycosylation mutant models to examine the effects of knockdown of specific genes involved in glycosylation. A knockout (fsck−/−) zebrafish model with mutations in the FCSK gene, which encodes fucokinase, an enzyme involved in fucosylation, exhibited neurodevelopmental defects along with locomotor deficiencies [9]. Further, zebrafish with phosphomannose isomerase (MPI) deficiency, an enzyme involved in the N-glycosylation of secretory proteins, showed multi-systemic deformities and increased embryonic lethality [10]. Notably, rescue was attained in the above-mentioned models with the supplementation of GDP-L-fucose or mannose, respectively [9,10].

Since all three of the basic N-glycan types are represented in zebrafish as early as 6 h post-fertilization (hpf), and are rich in oligomannose-type N-glycans [11], our prior studies included the generation of a GnT-I knockdown strain, specifically GnT-Ib, which resulted in diminished survivability, developmental delays, and aberrant spinal cord primary motor neuron structure relative to Wt AB zebrafish [12,13]. Since zebrafish have two GnT-I enzymes (GnT-Ia/b), unlike mice and humans, the study proved advantageous since the inactivation of *Mgat1* in neuronal tissue of mice yielded severe neurological defects and early post-natal death at approximately embryonic day (E13) [14], while global knockout of *Mgat1* in mice diminished survivability beyond (E10.5) due to maldevelopment of the neural tube [15]. Hence, knockdown of one of the GnT-I enzymes independent from the other in zebrafish was not lethal and prompted studies lasting into adulthood, which also set the stage for the next chapter of the project, the knockdown of GnT-Ia.

In this study, we created a *mgat1a* mutant fish model (*mgat1a*−/−) to compare along-side our previously generated *mgat1b*−/− model and the Wt AB strain. We showed that *mgat1a*−/−, like *mgat1b*−/−, has higher levels of oligomannose and less complex N-glycans compared to Wt AB zebrafish in all tissues tested, thus supporting a global effect. Additionally, we showed that *mgat1a*−/− has more oligomannose-type N-glycans than *mgat1b*−/− in all tested tissues, except brain of the zebrafish lines, and furthermore, different oligomannosylated protein expression patterns could be observed between *mgat1a*−/− and *mgat1b*−/−. Tail-coiling assays in embryos (24 hpf) showed motor function was most hampered in *mgat1b*−/− relative to Wt AB while *mgat1a*−/− was intermediate. Touch-evoked response assays established impaired motor and sensory functions in *mgat1a*−/− embryos (48 hpf) and larvae relative to Wt AB, which was previously reported in *mgat1b*−/− [12]. Likewise, dysfunctional locomotor activity propagated to adulthood, as the impaired swimming distance of adult fish was most pronounced in *mgat1b*−/− relative to Wt AB with *mgat1a*−/− intermediate. Anxiety-like behavior was modified in the *mgat1* mutant fish relative to Wt AB, but the mutant lines yielded opposite effects. Taken together, results of this study implicate that a reduction in complex-type N-glycans impedes the interaction between the nervous system and muscle to facilitate movement, as well as anxiety-like behavior.

## 2. Materials and Methods

### 2.1. Animal Husbandry, Larva and Embryo Collections

All zebrafish procedures received approval from the Institutional Animal Care & Use Committee (IACUC) at East Carolina University (AUP # C065b and approval date 29 January 2025). The adult wild-type (Wt) Pseudoloma-free AB strain, characterized by its robust health and suitability for research, was procured from the Sinnhuber Aquatic Research Laboratory, and subsequently propagated at ECU. This strain was utilized to develop mutant zebrafish through precise genetic editing of the *mgat1a* gene. Zebrafish were carefully maintained in a dedicated Pseudoloma-free, temperature-controlled environment (28 °C), adhering to a natural light/dark cycle of 14 h on and 10 h off. Embryos harvested from spontaneous spawning events were initially placed in 100 × 15 mm Petri dishes filled with egg water (5.03 mM NaCl, 0.17 mM KCl, 0.33 mM CaCl2•2H_2_O, 0.33 mM MgSO_4_•7H2O, and 0.05% methylene blue per liter of system water), and housed in the fish lab with water changes and feeding commenced twice daily, beginning at 5 days post-fertilization (dpf) to ensure optimal growth. At 5 dpf, larvae were transitioned to larger tanks with minimal egg water, and water exchanges commenced with a gradual change in the composition of the egg water relative to system water to allow for adequate acclimation (75:25 at 5 dpf; 50:50 at 6 dpf; 25:75 at 7 dpf; 0:100 at 8 dpf and beyond). All experiments utilizing live zebrafish were performed at 28 °C and used F2 and F3 generations of the mutant fish lines.

### 2.2. CRISPR/Cas9 Targets and Production of Purified sgRNA

The procedure used to engineer the *mgat1a*−/− mutant fish was like that previously described to create the *mgat1b*−/− [12]. In brief, the CHOPCHOP program was used to assign a guide RNA (gRNA) target sequence (CCCTGATCAGCGCAAAGACA) in zebrafish *Mgat1a* (Accession Number: NM_200676). The BclI site is underlined in the target sequence, which allowed for the identification of edited genomic DNA. The target sequence was designed with a T7 promoter sequence added to the 5′ end of the target sequence, as well as a 14 nucleotide overlap sequence to the 3′ end, for use with the EnGen^®^ sgRNA Synthesis Kit, S. pyogenes (New England Biolabs, Ipswich, MA, USA), followed by purification of the transcribed nucleotide via the Monarch^®^ Kit for RNA Cleanup (New England Biolabs).

### 2.3. Genotyping of Embryos, Larvae, and Adult Fish

Samples were collected (embryos, larvae, or a small portion of the adult fish tail) and genomic DNA extracted via incubation in 50 mM NaOH at 99 °C for 15–20 min. The supernatant containing DNA from the dissolved samples was used directly for PCR. PCR forward (agtacttcagagcgcttcatcc) and reverse (ggggcagttctacgacaagtac) primers were used to amplify the gRNA target region. PCR conditions were as previously reported [12]. Genetic modification of the fragment was ascertained by restriction enzyme (RE) digest, followed by band(s) separation on an agarose gel. An undigested band demonstrated that the DNA was edited for at least one of the *Mgat1a* alleles.

### 2.4. Engineering the mgat1a−/− Mutant Line

The *mgat1a*−/− mutant line was generated in a similar manner as the *mgat1b*−/− strain [12]. Single-cell embryos were microinjected with a 500-picoliter solution comprising 100 ng/μL of sgRNA and 360 pg/μL of EnGen Spy Cas9 NLS^®^. The microinjections were facilitated by compressed nitrogen gas and managed using a PV820 Pneumatic PicoPump (World Precision Instruments, Sarasota, FL, USA). A microcapillary pipette, connected to a micromanipulator, was employed under a Nikon microscope (Tokyo, Japan) for this process. To assess gRNA efficiency, at 24 hpf. about thirty microinjected embryos (F0) were collected, pooled, and genotyped. If the pooled F0 embryos displayed undigested bands after treatment with the restriction enzyme BclI (Thermofisher, Waltham, MA, USA), this indicated that Cas9 had successfully cleaved the targeted region, with the cell having incorrectly repaired the damage, thereby disrupting the BclI site and potentially resulting in a frameshift mutation. All remaining microinjected F0 embryos were raised to adulthood and later evaluated for gene editing through fin-clipping and restriction enzyme (RE) digestion. Moreover, adult F0 fish were outcrossed with Wt AB. F1 embryos were screened for gene editing via RE digestion, and then mutations were identified by DNA sequencing of the amplified DNA fragment. Male and female fish with identical mutations (△13) were crossed to generate F2 embryos and adult fish and screened by RE digestion.

### 2.5. Whole Brain and Brain Region Dissections

Prior to dissection of tissues, adult fish were humanely euthanized via anesthetization with MS222 followed by an ice slurry bath for 10 min until operculum movement ceased, in strict accordance with IACUC protocols, ensuring ethical treatment throughout the research process. For brain dissection, a similar protocol was followed to that outlined at https://app.jove.com/v/20201/zebrafish-brain-dissection-a-technique-of-fish-neurobiology (accessed on 12 February 2025). In summary, a euthanized fish was placed on a dissection bed and a surgical blade was used for decapitation at the level of the gill. Then, with the ventral side facing up, the soft tissues were removed using forceps. The optic nerves were severed with spring scissors, and the eyes were subsequently removed. Next, the fish was oriented with the dorsal side facing up, and portions of the skull were removed to isolate the brain, which was extracted using forceps. For brain sections, the whole brain was cut in the middle of the optic tectum. Region 1 of the brain includes the olfactory bulb, telencephalon, habenula, and the first half of the optic tectum. Region 2 is the second half of the optic tectum, cerebellum, and medulla. Whole brains and brain regions were placed in microcentrifuge tubes and stored at −80 °C until needed.

### 2.6. Dissections of Tissue

Zebrafish tissues were dissected as previously reported [13] and euthanization was carried out as outlined above. The spinal cord, heart, swim bladder, and skeletal muscle were collected. Dissected tissues were placed in cryotubes, flash-frozen in liquid nitrogen, and stored at −80 °C until ready for use.

### 2.7. Preparation of Homogenates

Adult zebrafish tissues were pooled (five to ten fish per tissue type) and collected for N-glycosylated protein analysis. Collected tissues were resuspended in RIPA buffer (PBS, 1% Triton X-100, 0.5% sodium deoxycholate, 0.1% SDS) plus protease inhibitor cocktail set III (EMD Biosciences, San Diego, CA, USA) and sonicated, followed by centrifugation. Supernatants were collected, followed by the addition of SDS-PAGE sample buffer containing DTT to reduce and denature the samples for lectin blotting and Coomassie blue staining.

### 2.8. Lectin Blots and Coomassie Blue-Stained Gels

The evaluation of proteins from tissue homogenate was conducted using Coomassie staining and lectin blotting techniques. The proteins were allowed to migrate on 10% SDS gels at 20 mA. Post migration, gels were either stained with Coomassie^®^ Brilliant Blue (MP Biomedical, Solon, OH, USA) or utilized to transfer proteins to a PVDF membrane (Whatman, Dassel, Germany) for lectin blotting, following previously established protocols [12]. The transferred proteins were then probed with *Galanthus nivalis* lectin (GNL lectin, Vector Laboratories, Burlingame, CA, USA). Image J 1.54d software was used for analysis of lectin blots and Coomassie blue-stained gels. Densitometric quantification of total band intensities per lane of lectin blots were normalized to total protein loading via Coomassie blue-stained gels, and then samples were normalized to *mgat1+/+* and Wt AB.

### 2.9. Tail-Coiling Assay

Embryos (24 hpf) [16] were individually placed into a 100 mm dish containing egg water and placed under a microscope for observation. An acclimation period of 1 min was followed by a 60 s testing period. The number of times the larvae’s tail coiled was measured through manual computation and the experimental procedure examined 50 embryos for Wt AB, *mgat1b*−/−, and *mgat1a+/*− fish strains, and 54 embryos for *mgat1a*−/−. During the procedure, it was noted that all embryos had heartbeats. After the procedure, the embryos were collected for genotyping. The data were collected blindly for *mgat1a*−/− and *mgat1a+/*−. In short, *mgat1a*−/− were crossed with *mgat1a+/*−, and then the tail-coiling assay was performed at 24 hpf. After assay, embryos were genotyped. The assay was performed on two separate days and in both cases about 50% of the embryos were *mgat1*−/− and the remainder was *mgat1a+/*−.

### 2.10. Swimming Locomotor Activity

Adult Wt AB (*n* = 17), *mgat1a*−/− (*n* = 37), and *mgat1b*−/− (*n* = 28) mutant fish were used to assay swimming distance with minor modifications, as previously described by Khotimah et al., 2015 [17]. In all cases, adult fish were of similar age and size. The vessel utilized was a 1.8 L tank (23 cm (L) × 5.5 cm (W) × 12 cm (H)), which had three vertical lines drawn on the bottom at equal distances of about 5.75 cm and contained 1.5 L of system water. The total distance swam correlated to the number of lines crossed. Each fish was placed into the tank and allowed to acclimate for five minutes, and then the number of lines crossed by the fish was counted for 5 min. Post recording, the fish was removed from the test tank and transferred to a separate tank. All fish were returned to the rack system following completion of the assay.

### 2.11. Novel Tank Dive Assay

The novel tank dive assay was used to assess anxiety-like behavior in adult fish of a similar age and size. A 1000 mL beaker with a line drawn at the 450 mL graduation mark to separate the beaker into lower and upper portions was used as the anxiety-inducing novel tank. Wt AB, the *mgat1b*−/− and the *mgat1a*−/− were individually placed in a beaker containing 1000 mL of system water and allowed five seconds to acclimate, followed by five minutes of observation of fish activity. Latency (time for fish to initially enter the upper chamber), number of times fish crossed the line to enter upper chamber, and total time spent in upper chamber were recorded. The number of fish used per strain was 20; 33 were used for Wt AB.

### 2.12. Touch-Evoked

A touch-evoked escape response assay was performed with embryos and larvae at 2 and 3 dpf for Wt AB (*n* = 40) and *mgat1a*−/− (*n* = 45). Fish were manually dechorionated using needles, if needed. After at least 1 h post dechorionation, 15 larvae were transferred to a 100 mm dish containing egg water and allowed to acclimate on a stereoscope for 10 min. A tactile stimulus was applied by a gentle touch to the tail of the larvae with a P10 micropipette tip. The escape behavior was tallied according to the number of touches it took before the larvae swam away (response).

### 2.13. Statistical Analysis

Adobe Photoshop was employed for agarose gel and lectin blot pictures. Origin 9.55 was used for graphics and statistics. A statistical comparison of two groups was accomplished using unpaired Student’s *t*-test and three or more groups were compared using one-way ANOVA with Bonholm’s adjustments. Statistical differences between data groups were also determined via Cohen’s *d* using Excel version 2502. Data are shown as the mean ± S.E. where *n* represents the number of observations, as indicated.

## 3. Results

### 3.1. Generating the mgat1a−/− Mutant Line

**Figure 1.**
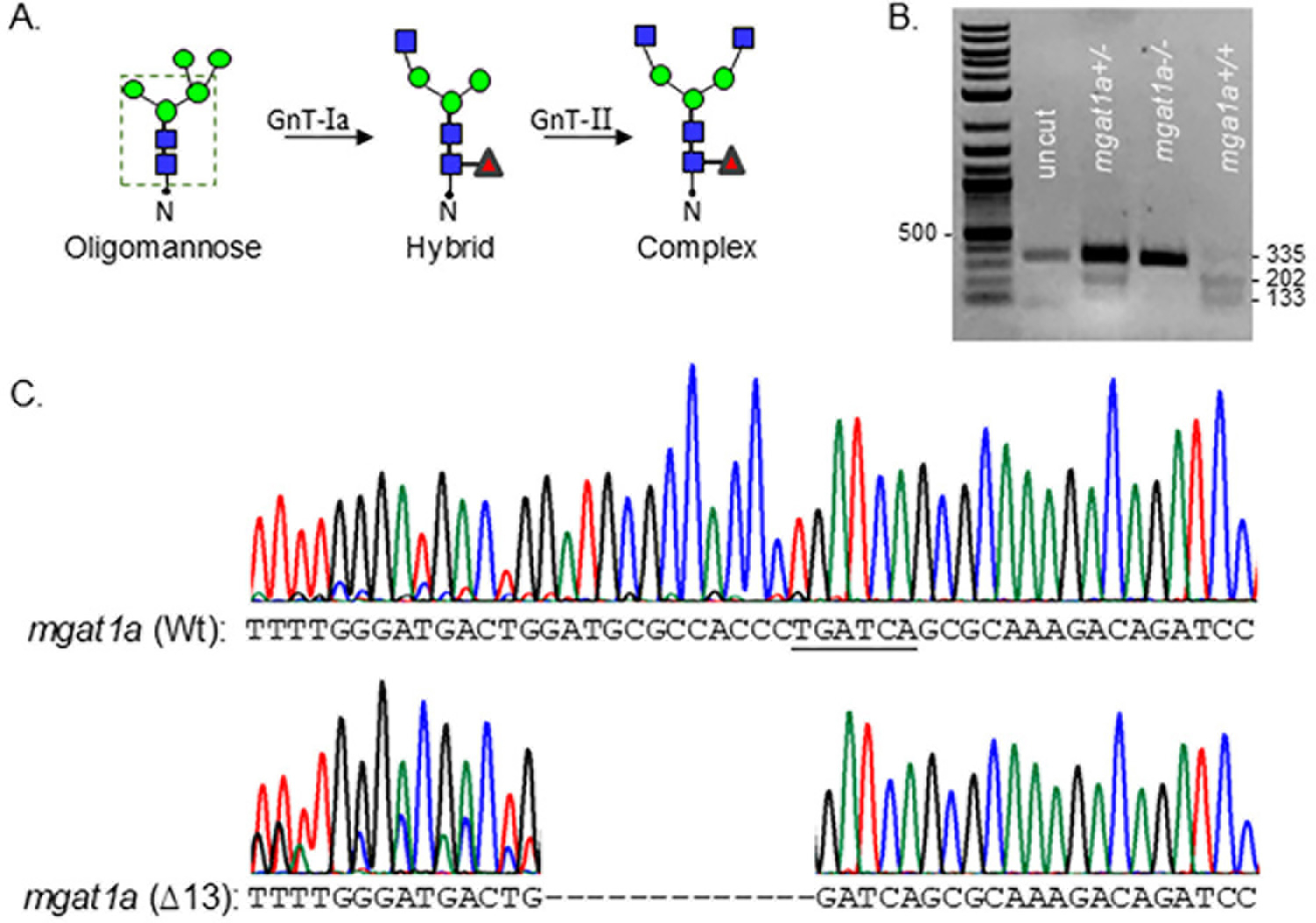
Characterization of the newly engineered *mgat1a*−/− zebrafish line. N-glycan types and the sequential processing initiated by GnT-I (*mgat1*) from oligomannose-type to hybrid-type and conversion of the latter type to complex-type via GnT-II (**A**). The dashed green box encloses the common pentasaccharide core. Blue, red, and green symbols denote GlcNAc, Fuc, and Man, residues, respectively. N represents Asn residue of an N-glycosylated protein. Agarose gel of genomic DNA fragment digested with BclI from heterozygous and homozygous fish raised from crossing *mgat1a+/*− fish with indel and Wt alleles (**B**). DNA Chromatogram of a fragment of *mgat1a* from a Wt AB fish compared to that of the *mgat1a*−/− fish, revealing thirteen nucleotide deletions (∆13) (**C**).

Zebrafish have two N-acetylglucosaminyl-tranferase-I (GnT-Ia and Ib) enzymes, while mice and humans have a single GnT-I [1,18]. GnT-I catalyzes the conversion of oligomannose-type N-glycans to hybrid-type N-glycans, which is subsequently converted to complex-type N-glycans via GnT-II (Figure 1A) [1]. Restriction enzyme digestion of an amplified fragment of the coding sequence (CDS) of *mgat1a* with an indel in the *mgat1a* were identified in adult fish (Figure 1B). Since the Wt AB allele contains a BclI site in the *mgat1a+/+*-amplified fragment, digestion of the fragment with BclI produced two bands, while the homozygous mutant fish (*mgat1a*−/−) produced a single larger band at a similar position as the uncut fragment. The fish that was heterozygous (*mgat1+/*−) for the mutation had three bands, signifying Wt and mutant *mgat1a* alleles. Thirteen nucleotides (∆13) were deleted in the *mgat1a* mutant zebrafish line, altering amino acid sequence from residue 299 onward and introducing a premature stop codon (Figure 1C). This deletion would disrupt the catalytic domain, which is localized in the C-terminal region [19], and thereby lower GnT-Ia activity.

### 3.2. Oligomannose N-Glycans Were Increased in the Homozygous mgat1a Mutant

**Figure 2.**
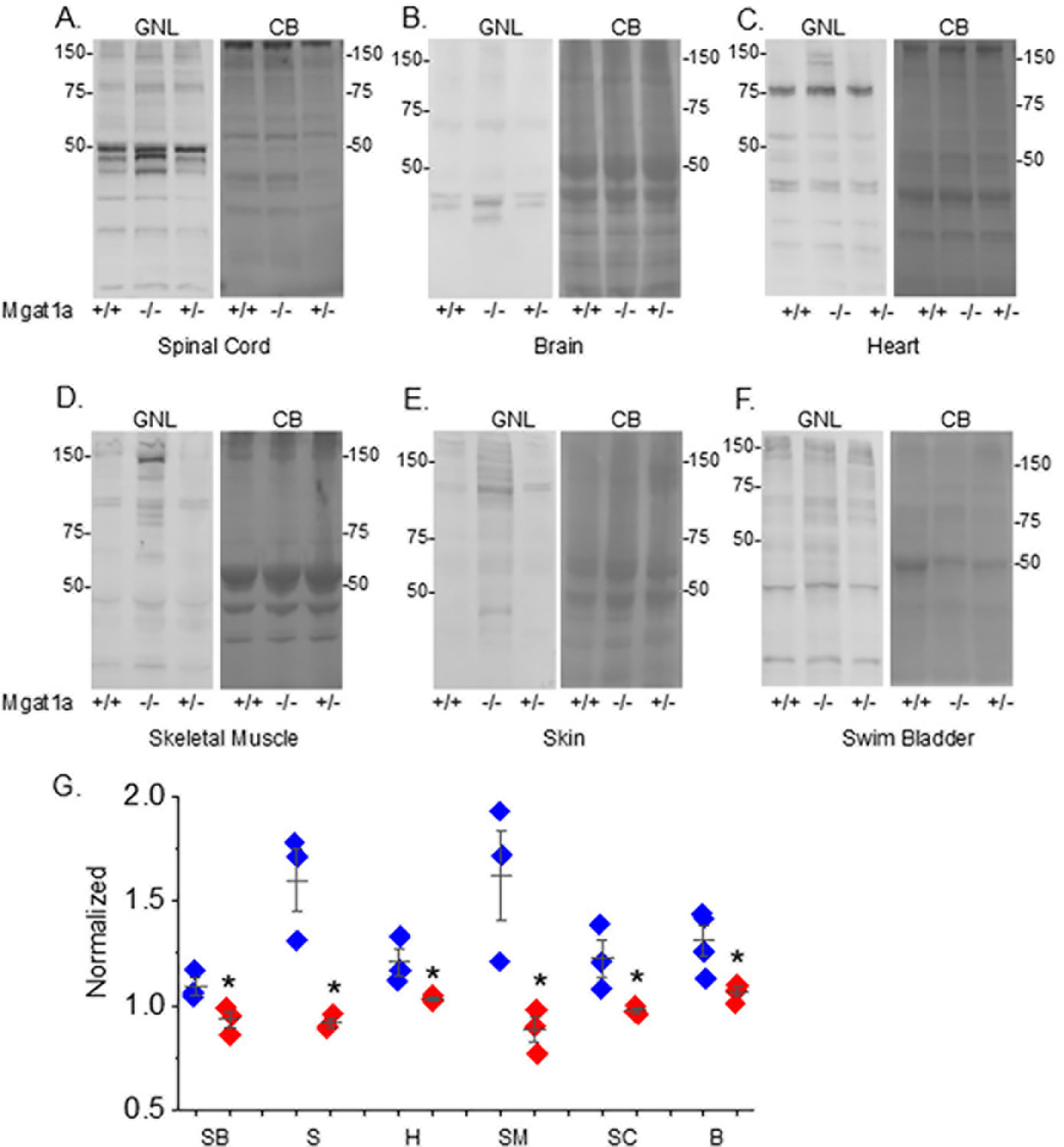
Increased levels of oligomannose-type N-glycans in adult *mgat1a*−/− zebrafish. Lectin blots of tissue homogenates from *mgat1a+/+*, *mgat1a*−/− and *mgat1a+/*− fish, as indicated (**A**–**F**). Electrophoresed proteins were probed with GNL (left panels). Protein gels stained with Coomassie blue (CB) (right panels) to demonstrate equal protein loads among the samples. Quantification of the oligomannosylated proteins from swim bladder (SB, *n* = 3), skin (S, *n* = 3), heart (H, *n* = 3), skeletal muscle (SM, *n* = 3), spinal cord (SC, *n* = 3), and brain (B, *n* = 4) (**G**). Numbers adjacent to the blots denote protein markers (in kDa). Data is mean ± S.E. and compared via Student’s *t*-test (* *p* at 0.05).

To examine the reduction in GnT-Ia activity in the *mgat1a* mutant fish, lectin blotting of spinal cord, brain, heart, skeletal muscle, skin, and swim bladder homogenates from fish with both Wt *mgat1a* alleles (*+/+*), both mutant alleles (−/−), and one of each of the two *mgat1a* alleles (*+/*−) was conducted (Figure 2). Glycosylated proteins from homogenates of each tissue were separated on a reducing SDS gel and probed with GNL. This lectin has higher affinity for oligomannose than hybrid or complex types of N-glycans [20]. In all cases, it was observed that the overall band intensities per lane from the various tissues were highest in the *mgat1a*−/− fish, while the *mgat1a+/+* and *mgat1a+/*− fish were quite similar to one another. In general, bands identified in *mgat1a+/+* and *mgat1a+/*− were of similar or darker intensity in *mgat1a*−/− but some new intense bands were detected in *mgat1a*−/− from the spinal cord, brain, heart, skeletal muscle, and skin. These results indicate that the homozygous mutant fish had higher levels of oligomannosylated protein, along with differences in oligomannosylated protein expression. Protein loads for each sample are shown on the accompanied coomassie blue (CB) gels. Of note, the levels of oligomannose N-glycans from tissues of Wt AB were much like those from the *mgat1a+/+* fish (Figure S1), indicating the absence of off-target effects in altering the N-glycosylation pathway. Quantification of the various tissues were conducted using additional lectin blots (Figure 2G) which can be viewed in Supplementary Materials. In all cases, the *mgat1a*−/− had significantly higher levels of oligomannosylated N-glycans. Thus, the higher levels of oligomannose N-glycans demonstrate that the indel in *mgat1a* reduced GnT-I activity, and that both copies of mutated *mgat1a* were required to increase levels of oligomannose N-glycans. Further raised levels of oligomannose N-glycans in six different tissues from the homozygous *mgat1a* mutant fish support that the reduction in the GnT-Ia activity was global.

### 3.3. Comparing Levels of Oligomannose N-Glycans Between mgat1a and mgat1b Mutant Fish

**Figure 3.**
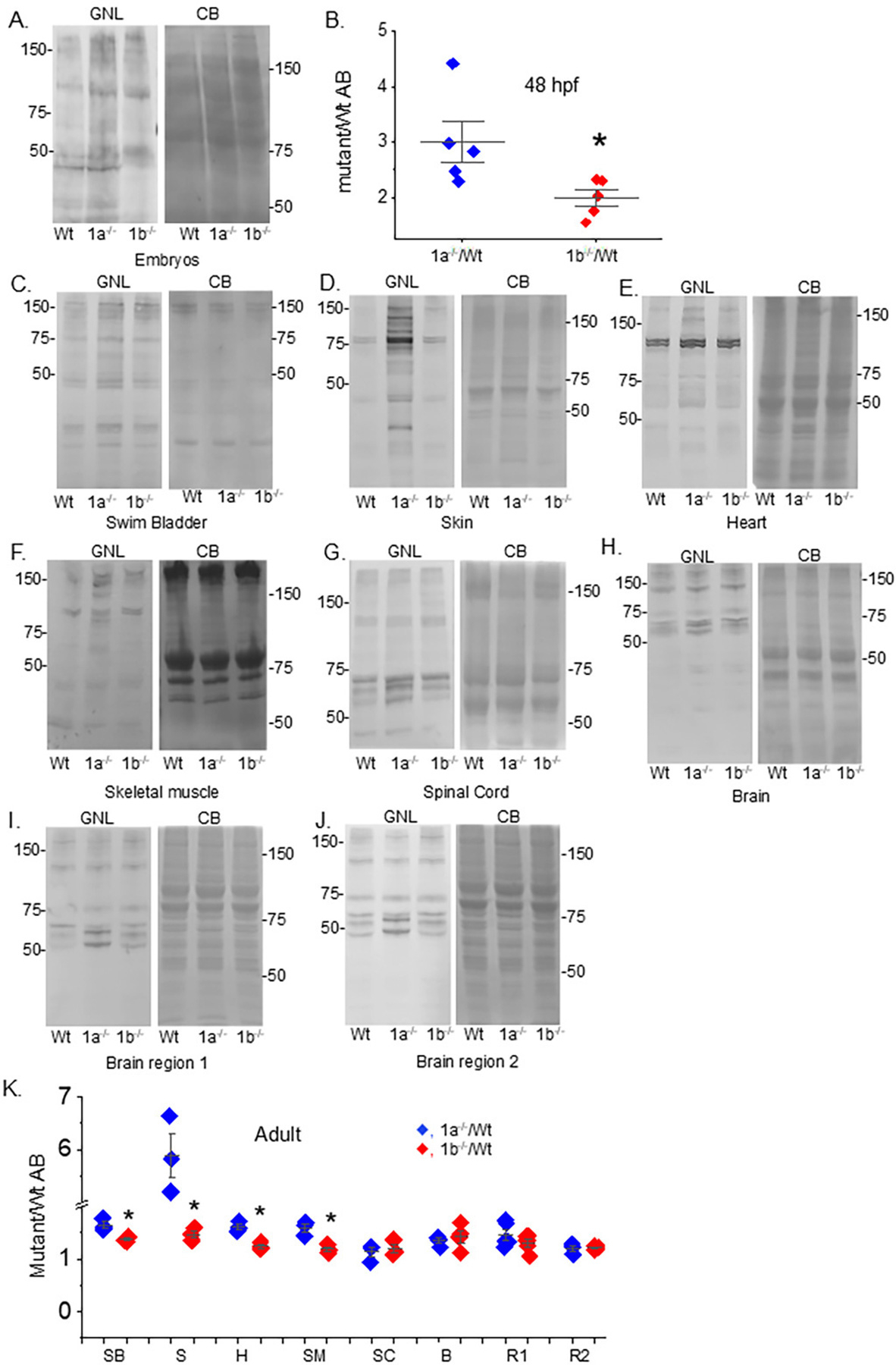
Comparison of oligomannosylated protein in mutant and Wt AB fish strains. Lectin blot and Coomassie blue-stained gels of embryo (48 hpf) (**A**), and tissues from adult strains (**C**–**J**). Quantification of the oligomannosylated proteins from embryos (*n* = 5; Panel (**B**)), and adult tissues, including swim bladder (SB, *n* = 3), skin (S, *n* = 3), heart (H, *n* = 3), skeletal muscle (SM, *n* = 3), spinal cord (SC, *n* = 3), brain (B, *n* = 4), brain regions 1 (R1, *n* = 5) and 2 (R2, *n* = 3) (Panel (**K**)). Brain regions 1 includes olfactory bulb, telencephalon, habenula, and about half of optic tectum, while brain region 2 includes about half of the optic tectum, cerebellum, and medulla. Numbers adjacent to the blots signify protein markers (in kDa). Data were compared Student’s t-test (* *p* at 0.02).

GNL blots of glycosylated protein of embryos (48 hpf), and also swim bladder, skin, heart, skeletal muscle, spinal cord, brain, brain region 1, and brain region 2 from adult Wt AB, *mgat1a*−/− and *mgat1b*−/− fish (Figure 3). Previously, the *mgat1b* mutant fish line was shown to have lowered GnT-I activity compared to Wt AB, resulting from the lowered expression of *mgat1b* [12]. Based on the visualized overall lane intensity, embryos from the *mgat1a*−/− fish line expressed the highest level of oligomannosylated proteins compared to Wt AB and *mgat1b*−/− (Figure 3A), which is significantly shown via quantification of lectin blots (Figure 3B). In adult fish, the *mgat1a*−/− fish had the highest level of oligomannosylated protein in the various tissues (Figure 3C–J). An exception was observed in spinal cord and brain, along with brain regions 1 and 2, as the *mgat1b*−/− had quite similar levels (Figure 3K). However, differences in the expression of oligomannosylated proteins were noted based on the different band patterns. Notably, the lowered portion of the blots had higher intensity bands for *mgat1a*−/− while the *mgat1b*−/− had higher intensity bands in the upper half of the blots. In all cases, it should also be noted that clear intense bands identified in the Wt AB were darkened in *mgat1b*−/− and *mgat1a*−/−. Thus, the lectin blots demonstrated that mutated *mgat1a* reduces GnT-I activity to a greater degree than mutated *mgat1b* in the tested tissues, except brain and spinal cord. Overall, they revealed that several complex/hybrid N-glycans associated with proteins in Wt AB were replaced with oligomannose N-glycans in the *mgat1* mutant lines. Further, the results supported that oligomannosylated protein patterns were different in all three fish lines.

### 3.4. Motor and Sensory Dysfunction in Embryos and Larvae with Lowered GnT-I Activity

**Figure 4.**
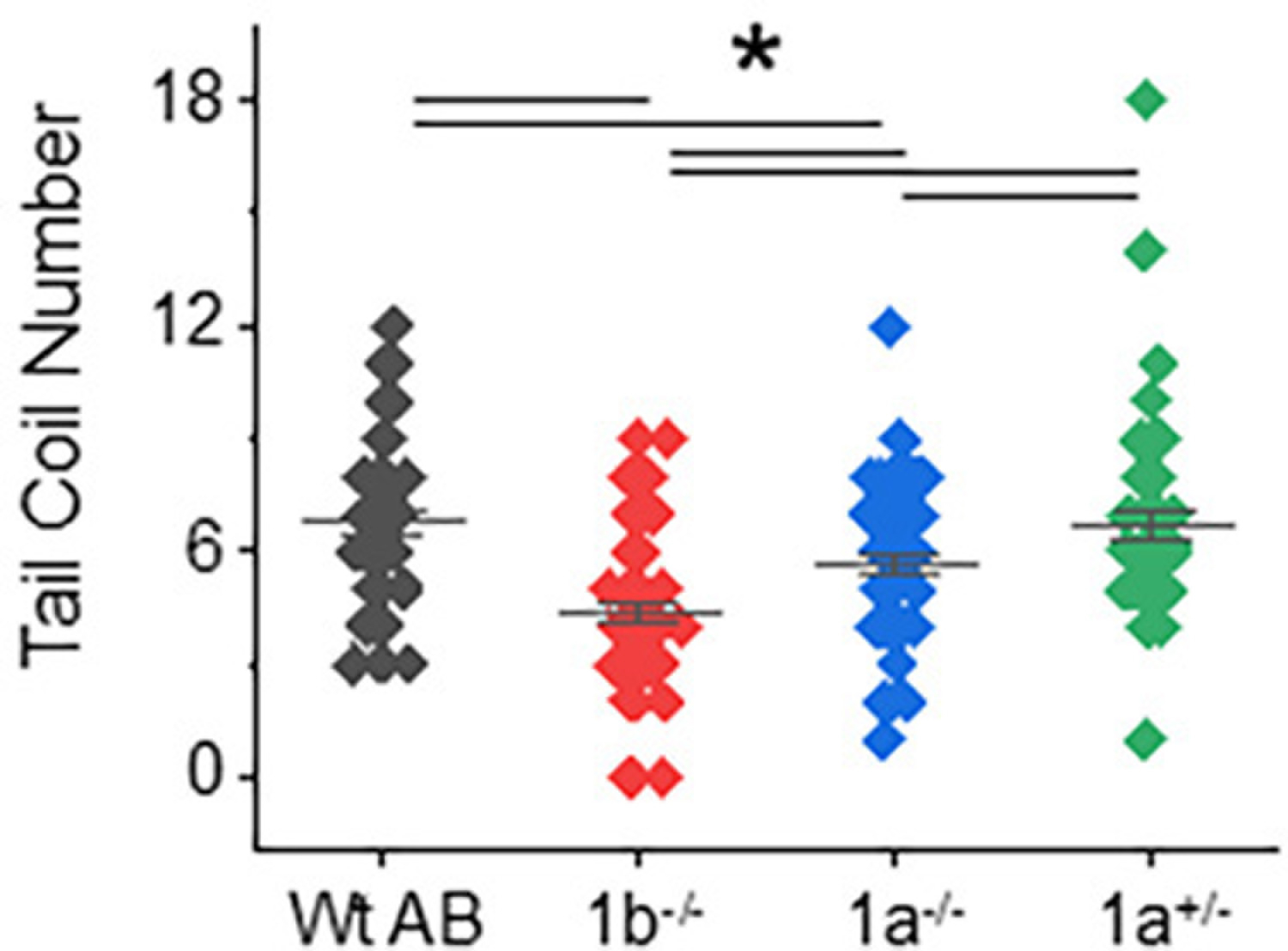
Side-by-side contractions were less frequent in the *mgat1* mutant embryos. The number of tail coils were recorded for a minute in Wt AB, *mgat1b*−/−, *mgat1a*−/−, and *mgat1a+/*− embryos (24 hpf). Data are presented on an interval scatter plot which includes the mean ± SEM, *n* = 50, except *n* = 54 for *mgat1a*−/−. Data were compared by one-way ANOVA with Bonholm test (* *p* at 0.05). Cohen’s *d* values revealed medium to large differences between the three groups as follows: Wt AB vs. *mgat1b*, 1.15; Wt AB vs. *mgat1a*−/−, 0.55; *mgat1a*−/− vs. *mgat1b*, 0.62. A difference between Wt AB and *mgat1a+/*− was lacking (*d =* 0.03) while large and medium differences were detected between *mgat1b*−/− vs. *mgat1+/*− (*d =* 1.0) and *mgat1a*−/− vs. *mgat1a+/*− (*d =* 0.45), respectively.

**Figure 5.**
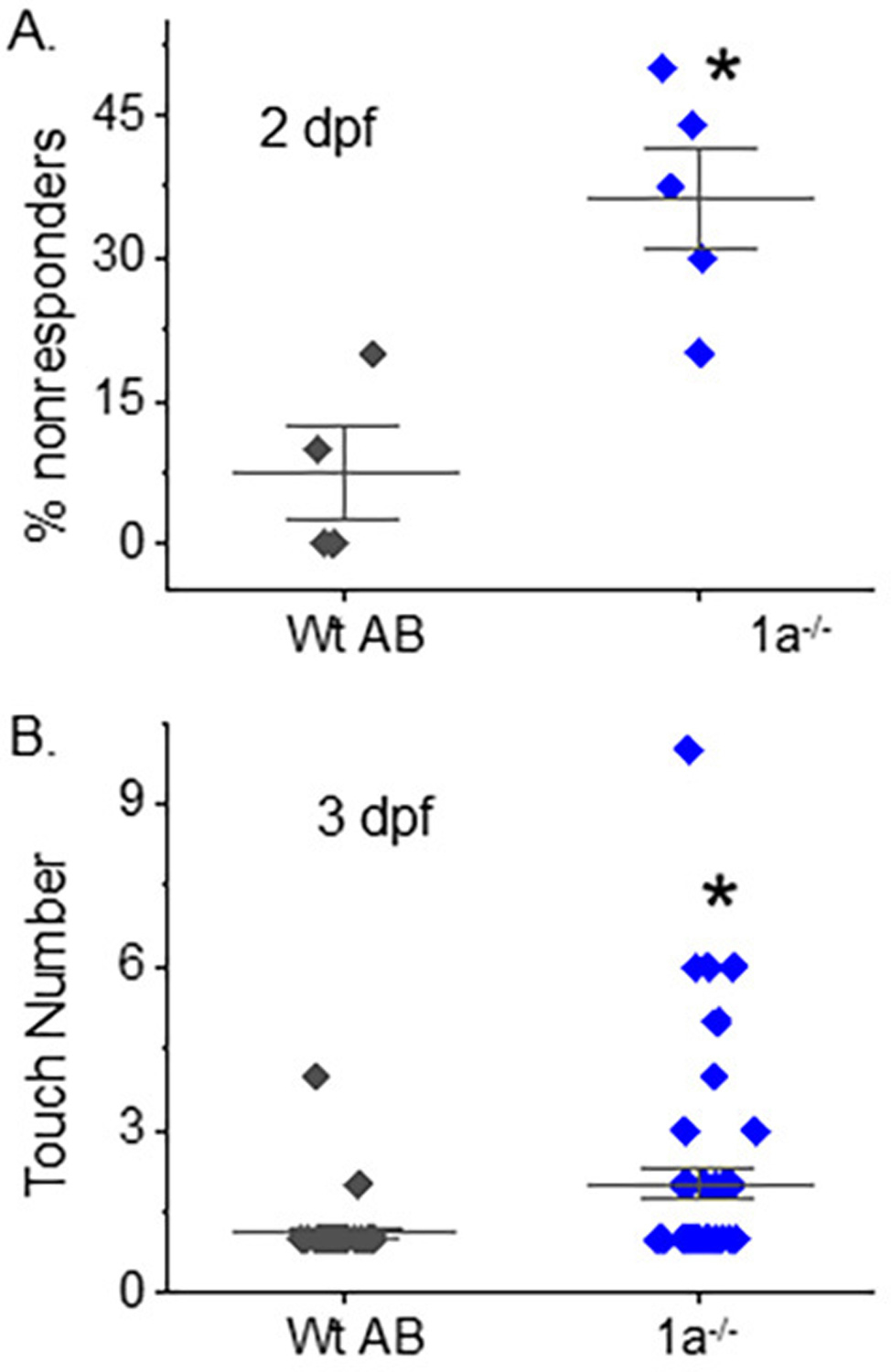
Maldevelopment of sensory and motor function in homozygous *mgat1* mutant embryo and larvae. Touch-evoked response assay of Wt AB and *mgat1a*−/− at 2 (**A**) and 3 dpf (**B**). The percentage of responders denotes number of embryos that were unresponsive to ten tail touches while the touch number is the number of touches it took for the embryo or larval to move. Data are presented as mean ± SEM, *n* = 4 (Wt AB), *n* = 5 (*mgat1a*−/−), where *n* denotes number of plates (**A**), and *n* = 40 (Wt AB) and *n* = 45 (*mgat1a*−/−), where *n* represents a fish (**B**), and were compared using Student’s t-test (* *p* < 0.006). Cohen’s *d* values supported medium to large differences between Wt AB and *mgat1a*−/− at 2 dpf (2.65) and 3 dpf (0.62).

To ascertain whether inhibiting terminal N-glycan processing affected motor behavior in the *mgat1* mutant fish lines, we employed spontaneous tail-coiling and touch-evoked response assays. Side-by-side contractions of the trunk were scored for Wt AB (6.8 ± 0.3, *n* = 50), *mgat1b*−/− (4.4 ± 0.3, *n* = 50), *mgat1a*−/− (5.7 ± 0.3, *n* = 54) and *mgat1a+/*− (6.7 ± 0.4, *n* = 50) embryos (24 hpf) (Figure 4). The *mgat1b*−/− mutant had the lowest number of tail coils and the Wt AB, like the *mgat1a+/*− mutant, had the highest tail coil number while the tail coil numbers of *mgat1a*−/− mutant were intermediate. Next, motor and sensory function were evaluated in more developed embryos (48 hpf) by determining whether the mutant embryos swam away when touched on the distal tail region. A high percent of the *mgat1a*−/− mutant embryos per plate (36.3 ± 5.3, *n* = 5) lacked a response to sense tactile stimuli while less than 10% of the Wt AB were unresponsive (Figure 5A). When evaluating larvae (72 hpf) in this manner, all larvae responded, but the response to the number of touches was higher for *mgat1a*−/− (2.0 ± 0.3, *n* = 44) than Wt AB (1.13 ± 0.08, *n* = 40) (Figure 5B). Notably, *mgat1a+/*− larvae had a similar response to touches as Wt AB, indicating that off-target effects on sensory and motor functions were absent; see Figure S2. Taken together, these results show that perturbation of the N-glycosylation pathway by decreasing the activity of GnT-I impaired motor and sensory functions in embryos and larvae from homozygous *mgat1* fish lines. Off-targets appeared void, as sensory and motor functions were like Wt AB.

### 3.5. Adult Fish with Increased Levels of Oligomannosylated Protein Have Deficient Motor Locomotion

**Figure 6.**
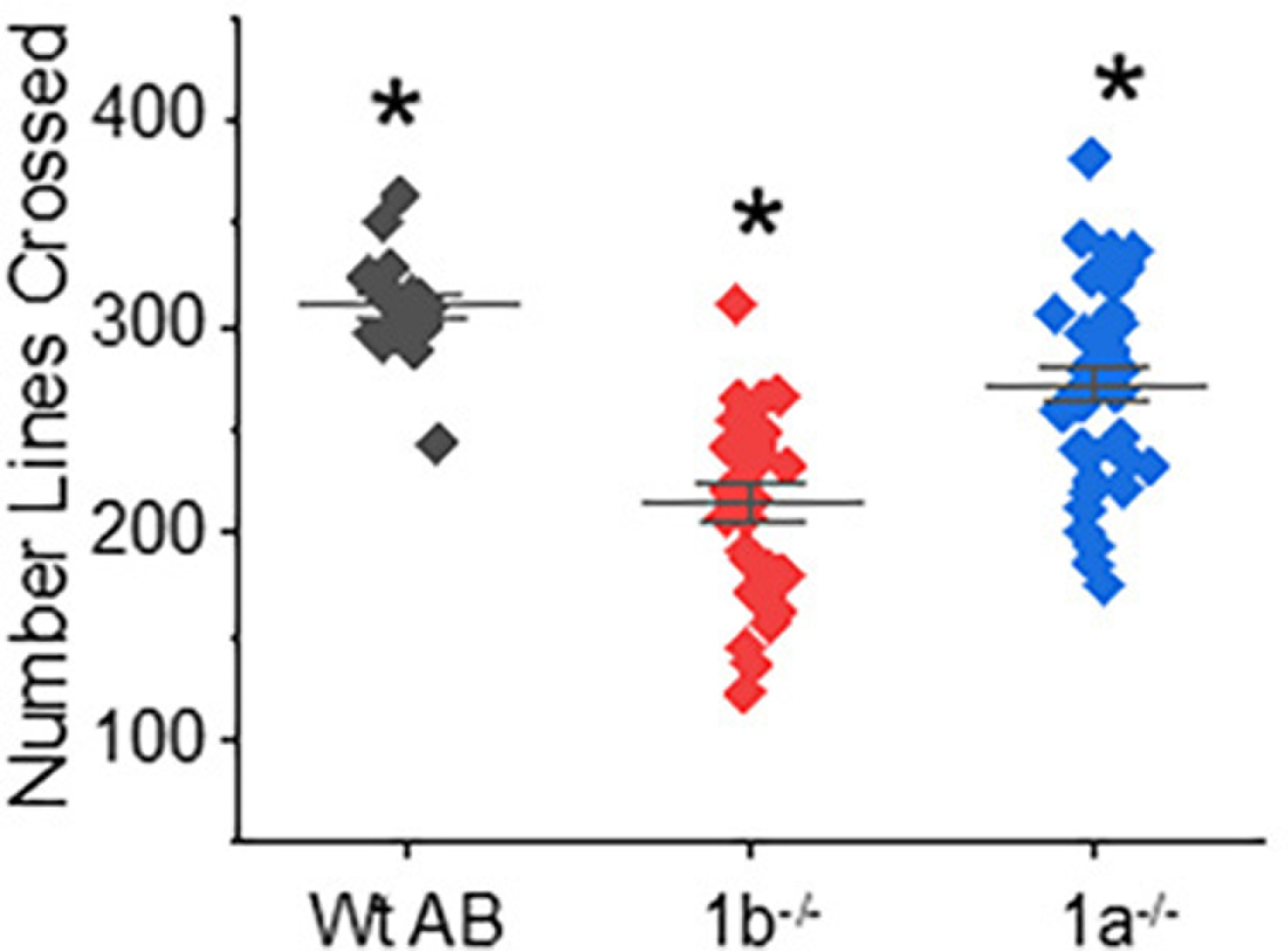
Swimming locomotor activity was declined in adult fish with defective GnT-I activity. The motor activity of Wt AB, *mgat1a*−/−, and *mgat1b*−/− fish was obtained by determining the total distance they could swim in five minutes. The total distance a fish swam was related to the number of lines crossed. Data are presented as mean ± SEM (n = 17, Wt AB; *n* = 28, *mgat1b*−/−; and *n* = 37, *mgat1a*−/−) and were compared using one-way Anova and Bonholm mean comparison test (* *p* at 0.001). Cohen’s *d* values between Wt AB and *mgat1b*−/− (2.42) and *mgat1a*−/− (0.89), as well as between the two mutant lines (1.19), were markedly different.

Since embryo and larvae had aberrant motility, swimming locomotor activity was evaluated in adult *mgat1* mutant fish lines. The normal behavior of a fish is to swim back and forth along the length of the tank, so the total distance a fish could swim was reported by the number of times a fish crossed a line in five minutes [17]. The total swimming distance for adult fish from the *mgat1* mutant strains (*mgat1b*−/−, 215 ± 9, *n* = 28); *mgat1a*−/−, 272 ± 8, *n* = 37) was significantly less than those from the Wt AB line (310 ± 6, *n* = 17) (Figure 6). Moreover, a shorter distance was observed for the *mgat1b*−/− fish than the *mgat1a*−/− fish. Hence, dysfunction of locomotor activity recorded during the development of the mutant fish was perpetuated in the adult fish.

### 3.6. Reduced Terminal N-Glycan Processing Alters Anxiety-like Behavior

**Figure 7.**
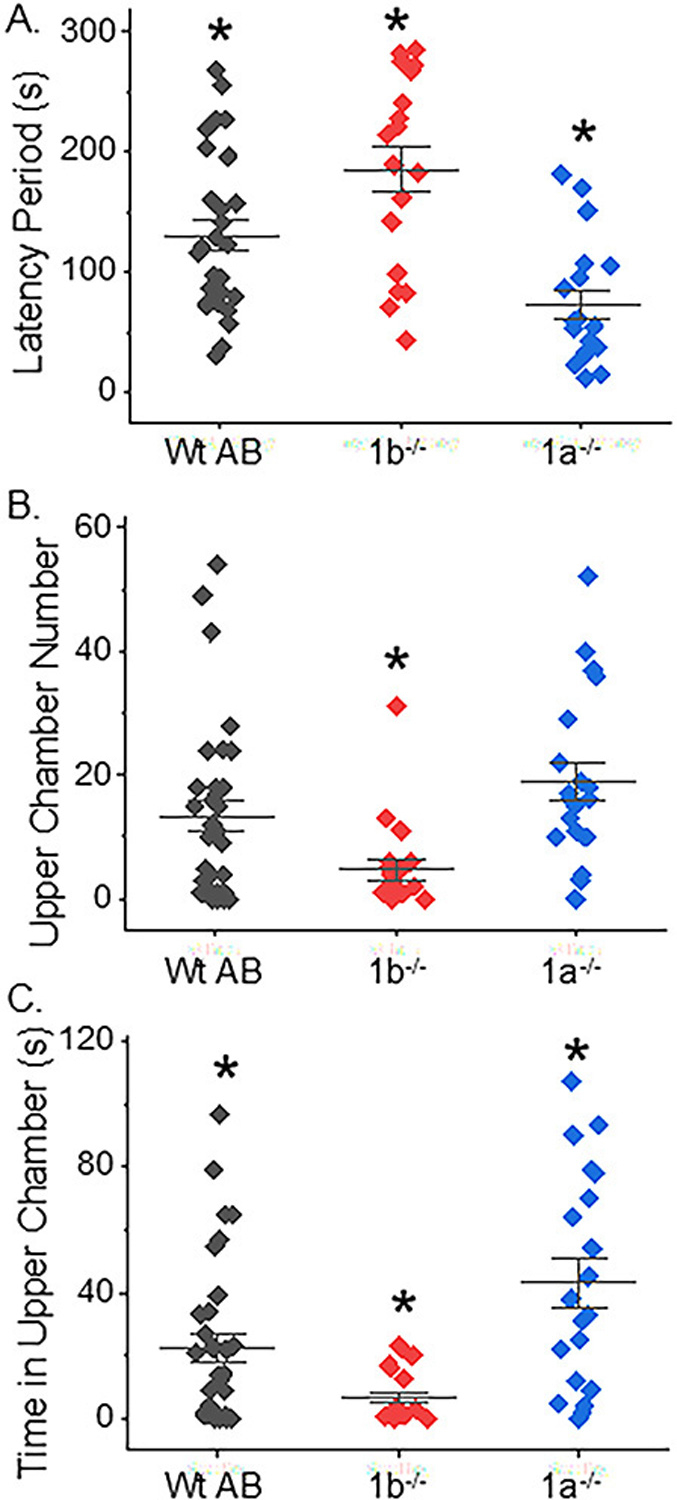
Anxiety-type behavior was aberrant in adult *mgat1* mutant fish. Computations of the time taken for fish to swim to the upper chamber of the tank (latency), the total number of times the fish moved from the lower to upper chamber, and the amount of time spent in the upper chamber were all measured in efforts to fully examine the impact of anxiety-type behavior in zebrafish. Data show results for each fish and the mean ± SEM for each measured parameter (*n* = 28, Wt AB; *n* = 18, *mgat1b*−/−; and *n* = 19, *mgat1a*−/−) (**A**) and (*n* = 33, Wt AB; *n* = 20, *mgat1b*−/−/; and *n* = 20, *mgat1a*−/−) (**B**,**C**). Data were compared using one-way Anova, along with Bonholm mean comparison test (* *p* < 0.05). Cohen’s *d* values signified medium to large differences between the three groups, as follows: WtAB vs. mgat1b, 0.76, 0.71, 0.73; Wt AB vs. mgat1a, 0.94, 0.40, 0.71; mgat1a vs. mgat1b, 1.68, 1.31, 0.1.46 for latency period, upper chamber number, and time in upper chamber, (**B**), and (**C**), respectively.

The novel tank diving test was employed to evaluate anxiety-like behavior in fish. The results show the time it took for an adult fish to move from the bottom half of beaker to the upper half of beaker, which is referred to as the latency period (Figure 7A). The latency period was longer for the *mgat1b*−/− fish (186 ± 19, *n* = 18) and shorter for the *mgat1a*−/− fish (73 ± 12, *n* = 19) when compared to Wt AB fish (130 ± 13, *n* = 28). The number of times the fish moved from the bottom half (lower chamber) to top half (upper chamber) of the beaker was scored (Figure 7B). The number of these transitions was slightly more altered for the *mgat1a*−/− fish (20 ± 3, *n* = 20) compared to Wt AB (16 ± 3, *n* = 33), while the effect was quite large for the *mgat1b* fish (5 ± 2, *n* = 20). The total time the fish resided in the upper chamber was significantly greater for *mgat1a*−/− (45 ± 8, *n* = 20) than Wt AB (26 ± 5, *n* = 33), while it was greatly reduced for *mgat1b*−/− (7 ± 2, *n* = 20) (Figure 7C). Of note, fish stayed close to the wall upon their entrance into the tank; however, the fish were less likely to localize in proximity to the wall upon movement to top chamber, which was particularly noted for the *mgat1a*−/− fish (personal observation). Taken together, these results demonstrate that anxiety-like behavior is altered in the *mgat1* mutant fish relative to Wt AB fish but the increased oligomannosylated protein in the two mutant lines has opposing effects on anxiety levels relative to Wt AB.

## 4. Discussion

Previously, our lab established that embryo and larvae zebrafish had decreased survivability, delayed development, and deficient sensory and motor function when one (GnT-Ib) of the GnT-I enzymes (GnT-Ia/b) was knocked out [12,13]. Here, our research was broadened to assess the expression pattern and harmful consequences of a global reduction in GnT-Ia in zebrafish, and furthermore to compare the mutant *mgat1a* and *mgat1b* zebrafish models, particularly in adult fish. A comparison of *mgat1a+/*− to *mgat1a*−/− fish lines revealed similarities in embryonic survivability, onset of heartbeat, and embryonic and larvae motor and sensory functions, indicating that off-target effects were virtually absent. Increased levels of oligomannose were detected in embryos and various dissected tissues of adult fish when the expression of *mgat1a* was reduced, which also occurred in the *mgat1b* mutant zebrafish model [12,13]. These results indicated that the *mgat1a*/b genes do not fully compensate for each other. In a direct comparison of the *mgat1* mutant models, we observed that oligomannose levels were higher in spinal cord, skeletal muscle, heart, skin, and swim bladder for the *mgat1a* fish, while they were quite similar in brain. These increases in oligomannose N-glycans correspond with decreases in complex N-glycans, as established by the down-regulation of the *mgat1b* zebrafish model [12,13], the *Mgat1* mice models [14,15], and *Mgat1* in neuroblastoma (NB) [21,22] and Chinese hamster ovary (CHO) [23] cell lines. Dissimilar band patterns were also observed on lectin blots of *mgat1a* and *mgat1b*, supporting differences in the expression of oligomannosylated proteins. Our results indicated that *mgat1a* was more widely expressed than *mgat1b* in adult fish, as well as embryos. A similar finding was observed for *mgat1a*/b expression data during the development of zebrafish [18]. Next, it was shown that motor function was more deficient in embryo, larvae, and adult *mgat1b* fish. Further the *mgat1b* displayed the strongest anxiety-like behavior and *mgat1a* fish had the lowest anxiety, while Wt AB had an intermediate level. Taken together, these results support that the replacement of complex/hybrid N-glycans with oligomannose N-glycans on proteins in the two *mgat1* zebrafish models had different effects. Moreover, the changes in N-glycan populations of both *mgat1* models have differing impacts on motor function and anxiety-like behavior.

Regarding the *Mgat1* mouse model, there are implications for neuronal and locomotor malfunction [14]. Further when some of the complex-type N-glycans were substituted with oligomannose-type N-glycans in the *mgat1b* mutant fish, the spinal cord caudal primary motor (CaP) neurons were shown to be poorly developed up to 76 hpf, and delays in muscle development were evident [12,13]. Since *mgat1a* mutant fish have decreased complex-type N-glycans, like the *mgat1b* mutant fish, we anticipate that the CaP neurons in the *mgat1a* mutant fish have maldeveloped CaP neurons. Delays in neuron and muscle development are supported by the deficiency in motor activity, as spontaneous tail-coiling of the embryos is facilitated by axial muscle innervation by the primary motor neurons [24,25]. Defects in motor and sensory functions were also evident based on touch-evoked response assays. CaP neurons, along with other primary motor neurons, innervate the ventral musculature to mediate the large-amplitude muscle contraction by tactile stimuli [26–29]. Our data also showed that deficient motor activity and altered anxiety-like behavior persisted into adulthood. Thus, maldevelopment of muscular and spinal control, along with brain function, occur due to perturbations in terminal N-glycan processing by either GnT-Ia or GnT-Ib.

The interaction between the nervous system and muscle to enable movement was defective in the *mgat1* zebrafish lines. The innervation of the ventral musculature by the spinal cord primary motor neurons was more delayed in the *mgat1b*−/− than the *mgat1a*−/− embryos, as indicated by the spontaneous tail-coiling assay. In comparing the touch-evoked response of the two fish lines, the number of *mgat1b* [12] and *mgat1a* embryos (2 dpf) lacking response to touch was quite similar, while the number of non-responders among the *mgat1b* larvae [12] was greater than that among the *mgat1a* larvae (3 dpf). A comparison of *mgat1a* and *mgat1b* transcript levels further supports that the *mgat1b* mutant fish are more deficient in embryonic motor activity, along with the motor and sensory functions of embryos and larvae. For instance, the cholinergic-enriched motor neurons of the spinal cord express much higher levels of *mgat1b* transcripts than *mgat1a* from 14 to 82 hpf [18]. The sensory neurons of the dorsal spine express both *mgat1a* and *mgat1b* from 48 to 82 hpf, with the expression of *mgat1b* being higher from 72 to 82 hpf [18]. Furthermore, the brain has elevated expression levels of *mgat1b* relative to *mgat1a* in the hind brain and dienephalon–tuberculum, which are essential for sensory and motor functions [18,30,31]. Additionally, deficiencies in the motor activity of adult *mgat1* mutant fish were observed, with a more defects in *mgat1b* fish, indicating that impaired muscular, brain, and spinal control persisted into adulthood for both *mgat1a* and *mgat1b* mutant fish, with more detrimental effects on the *mgat1b*−/− fish. Hence, we suspect that decreases in the activity of GnT-I would contribute to neurodevelopmental disorders.

Anxiety-like behavior was decreased and increased in *mgat1a*−/− and *mgat1b*−/− fish, respectively, compared to Wt AB. This was shown using the novel tank dive assay as *mgat1a*−/− fish made their first entrance to the upper chamber in the shortest time and resided in the upper chamber for the longest time, while the *mgat1b*−/− took the longest time to swim to the upper chamber and spent the least amount of time in the upper chamber. The adult *mgat1b*−/− zebrafish showed significant alterations to glycan profiles of the brain relative to Wt AB [12]. Moreover, the lectin blots supported increases in different oligomannosylated proteins of brain between *mgat1b*−/− and *mgat1a*−/− zebrafish. This difference is quite apparent in brain region 1, which includes the region recently identified as being equivalent to the human amygdala, responsible for fear and anxiety behaviors [32,33]. Stress response has also been related to large tyrosine hydroxylase dopaminergic neurons of the paraventricular nucleus [31]. Taken together, it may be that these differing impacts on anxiety-like behavior are related to GnT-I a/b activity in different areas of the brain.

CDG, an ever-growing group of metabolic diseases caused by underglycosylation and aberrant terminal N-glycosylation processing, is multisystemic, and frequently causes developmental delays, ataxia, psychomotor retardation, and anxiety [3–7]. The *mgat1a/b* mutant zebrafish models provide a feasible route to directly address how reduced terminal N-glycosylation processing can affect various organs. Since CDG patients often have deficient motor activity, it would be of interest to reintroduce *mgat1a/b* expression in spinal cord primary motor neurons of the *mgat1a/b* zebrafish models. Further, the role of Kv3 channels in spinal cord primary motor neurons of the various models should be evaluated, as Kv3 channels are critical components of these neurons [34,35] and disruptions in Kv3 expression and activity are associated with aberrant axonal pathfinding [36] and deficiencies in locomotor activity [34,35].

## 5. Conclusions

Disrupted N-glycan processing is a known factor involved in neurodegenerative and neurological disorders [5,8], as well as the ever-growing field of congenital disorders of glycosylation (CDG). Due to the limited treatment options available for patients impacted by these disorders, glycans are appealing therapeutic targets. Our study using glycosylation mutant (*mgat1*) zebrafish exposes how the substitution of complex/hybrid with oligomannose types of N-glycans relates to the interaction between the nervous and musculature systems. While the motor and sensory functions of embryos and larvae from both *mgat1* strains were deficient and remained impaired into adulthood, the impacts were different among the strains and are quite in line with transcript levels. Moreover, modifications in anxiety-like behaviors further demonstrated the impact of the exchange between the various types of N-glycans on the nervous system. Specifically, it is possible that variability in oligomannosylated proteins in the brain, spinal cord, and muscle are related to certain anxiety-related behaviors, and deficiencies in the motor and sensory functions. Further investigation could aim to restore *mgat1a/b* in certain cells of brain, spinal cord, and skeletal muscle of the *mgat1* mutant models. We conclude that decreases in the activity of GnT-I contributes to nervous system development and maintenance and, as such, is worthy of further exploration to improve treatment opportunities.

## Supplementary Material

Supplementary Material

The following supporting information can be downloaded at: https://www.mdpi.com/article/10.3390/ijtm5030036/s1. Figure S1: Similar levels of oligomannose in Wt AB and mgat1a+/+. Lectin blot and Coomassie blue-stained gels from tissues, as indicated (A–F) to illustrate that levels of oligomannose N-glycans from tissues of Wt AB were like those from the *mgat1a+/+* fish, indicating the absence of off-target effects in altering the N-glycosylation pathway. Lines adjacent to the blots denote protein markers (in KDa) 250, 150, 100, 75, 50, 37. Figure S2: Touch evoked response of Wt AB and *mgat1a*−/− larvae at 3 dpf. Five larvae were added to a 100 mm dish, and allowed to acclimate for 3 min, prior to touching posterior end of tail. Data are presented as mean ± SEM, *n* = 51 and 59 for Wt AB and *mgat11a+/*−, respectively. Data were compared by student t-test (* *p* < 0.05).

## Data Availability

The original contributions presented in this study are included in the article/Supplementary Materials. Further inquiries can be directed to the corresponding author.

## Abbreviations

The following abbreviations are used in this manuscript:

GNL: Galanthus nivalis lectin
GnT: N-acetylglucosaminyltransferase
DPF: Days post-fertilization
HPF: Hours post-fertilization
CDG: Congenital disorders of glycosylation
Wt: Wild-type

## References

[1] Stanley P; Moremen KW; Lewis NE; Taniguchi N; Aebi M N-Glycans. Essentials of Glycobiology; Varki A, Cummings RD, Esko JD, Stanley P, Hart GW, Aebi M, Mohnen D, Kinoshita T, Packer NH, Prestegard JH, , Eds.; Cold Spring Harbor Laboratory Press: New York, NY, USA, 2022.

[2] Sosicka P; Ng BG; Freeze HH Chemical Therapies for Congenital Disorders of Glycosylation. ACS Chem. Biol 2022, 17, 2962–2971. 34788024 10.1021/acschembio.1c00601 PMC9126425

[3] Jaeken J Congenital disorders of glycosylation. Ann. N. Y. Acad. Sci 2010, 1214, 190–198. 21175687 10.1111/j.1749-6632.2010.05840.x

[4] Ondruskova N; Cechova A; Hansikova H; Honzik T; Jaeken J Congenital disorders of glycosylation: Still “hot” in 2020. Biochim. Biophys Acta Gen. Subj 2021, 1865, 129751. 32991969 10.1016/j.bbagen.2020.129751

[5] Pradeep P; Kang H; Lee B Glycosylation and behavioral symptoms in neurological disorders. Transl. Psychiatry 2023, 13, 154. 37156804 10.1038/s41398-023-02446-x PMC10167254

[6] Verheijen J; Tahata S; Kozicz T; Witters P; Morava E Therapeutic approaches in Congenital Disorders of Glycosylation (CDG) involving N-linked glycosylation: An update. Genet. Med 2020, 22, 268–279. 31534212 10.1038/s41436-019-0647-2 PMC8720509

[7] Van de Loo KFE; van Dongen L; Mohamed M; Gardeitchik T; Kouwenberg TW; Wortmann SB; Rodenburg RJT; Lefeber DJ; Morava E; Verhaak CM Socio-emotional Problems in Children with CDG. JIMD Rep 2013, 11, 139–148. 23733602 10.1007/8904_2013_233 PMC3755554

[8] He M; Zhou X; Wang X Glycosylation: Mechanisms, biological functions and clinical implications. Signal Transduct. Target. Ther 2024, 9, 194. 39098853 10.1038/s41392-024-01886-1 PMC11298558

[9] Liu ZX; Zou TT; Liu HH; Jia HB; Zhang XQ Knockout of the fcsk gene in zebrafish causes neurodevelopmental defects. Zool. Res 2025, 46, 313–324. 40049660 10.24272/j.issn.2095-8137.2024.229 PMC12000135

[10] Chu J; Mir A; Gao N; Rosa S; Monson C; Sharma V; Steet R; Freeze HH; Lehrman MA; Sadler KC A zebrafish model of congenital disorders of glycosylation with phosphomannose isomerase deficiency reveals an early opportunity for corrective mannose supplementation. Dis. Models Mech 2013, 6, 95–105. 10.1242/dmm.010116 PMC3529342 22899857

[11] Hanzawa K; Suzuki N; Natsuka S Structures and developmental alterations of *N*-glycans of zebrafish embryos. Glycobiology 2017, 27, 228–245. 27932382 10.1093/glycob/cww124

[12] Hall MK; Hatchett CJ; Shalygin S; Azadi P; Schwalbe RA Reduction in *N*-Acetylglucosaminyltransferase-I Activity Decreases Survivability and Delays Development of Zebrafish. Curr. Issues Mol. Biol 2023, 45, 9165–9180. 37998752 10.3390/cimb45110575 PMC10669939

[13] Hatchett CJ; Hall MK; Messer AR; Schwalbe RA Lowered GnT-I Activity Decreases Complex-Type *N*-Glycan Amounts and Results in an Aberrant Primary Motor Neuron Structure in the Spinal Cord. J. Dev. Biol 2024, 12, 21. 39189261 10.3390/jdb12030021 PMC11348029

[14] Ye Z; Marth JD *N*-glycan branching requirement in neuronal and postnatal viability. Glycobiology 2004, 14, 547–558. 15044398 10.1093/glycob/cwh069

[15] Ioffe E; Stanley P Mice lacking *N*-acetylglucosaminyltransferase I activity die at mid-gestation, revealing an essential role for complex or hybrid N-linked carbohydrates. Proc. Natl. Acad. Sci. USA 1994, 91, 728–732. 8290590 10.1073/pnas.91.2.728 PMC43022

[16] Selderslaghs IW; Hooyberghs J; De Coen W; Witters HE Locomotor activity in zebrafish embryos: A new method to assess developmental neurotoxicity. Neurotoxicol. Teratol 2010, 32, 460–471. 20211722 10.1016/j.ntt.2010.03.002

[17] Khotimah H; Ali M; Sumitro SB; Widodo MA Decreasing α-synuclein aggregation by methanolic extract of Centella asiatica in zebrafish Parkinson’s model. Asian Pac. J. Trop. Biomed 2015, 5, 948–954.

[18] Sur A; Wang Y; Capar P; Margolin G; Prochaska MK; Farrell JA Single-cell analysis of shared signatures and transcriptional diversity during zebrafish development. Dev. Cell 2023, 58, 3028–3047.e12. 37995681 10.1016/j.devcel.2023.11.001 PMC11181902

[19] Sarkar M; Pagny S; Unligil U; Joziasse D; Mucha J; Glossl J; Schachter H Removal of 106 amino acids from the N-terminus of UDP-GlcNAc: Alpha-3-D-mannoside beta-1,2-N-acetylglucosaminyltransferase I does not inactivate the enzyme. Glycoconj. J 1998, 15, 193–197. 9557881 10.1023/a:1006928624913

[20] Patnaik SK; Stanley P Lectin-resistant CHO glycosylation mutants. Methods Enzymol 2006, 416, 159–182. 17113866 10.1016/S0076-6879(06)16011-5

[21] Hall MK; Burch AP; Schwalbe RA Functional analysis of *N*-acetylglucosaminyltransferase-I knockdown in 2D and 3D neuroblastoma cell cultures. PLoS ONE 2021, 16, e0259743. 34748597 10.1371/journal.pone.0259743 PMC8575246

[22] Burch AP; Kristen Hall M; Wease D; Schwalbe RA Reduction of *N*-Acetylglucosaminyltransferase-I Activity Promotes Neuroblastoma Invasiveness and EGF-Stimulated Proliferation In Vitro. Int. J. Transl. Med 2024, 4, 519–538. 10.3390/ijtm4030035 PMC11687401 39742082

[23] North SJ; Huang H-H; Sundaram S; Jang-Lee J; Etienne AT; Trollope A; Chalabi S; Dell A; Stanley P; Haslam SM Glycomics profiling of Chinese hamster ovary cell glycosylation mutants reveals *N*-glycans of a novel size and complexity. J. Biol. Chem 2010, 285, 5759–5775. 19951948 10.1074/jbc.M109.068353 PMC2820803

[24] Kimmel CB; Patterson J; Kimmel RO The development and behavioral characteristics of the startle response in the zebra fish. Dev. Psychobiol 1974, 7, 47–60. 4812270 10.1002/dev.420070109

[25] Saint-Amant L; Drapeau P Time course of the development of motor behaviors in the zebrafish embryo. J. Neurobiol 1998, 37, 622–632. 9858263 10.1002/(sici)1097-4695(199812)37:4<622::aid-neu10>3.0.co;2-s

[26] Liu DW; Westerfield M Function of identified motoneurones and co-ordination of primary and secondary motor systems during zebra fish swimming. J. Physiol 1988, 403, 73–89. 3253426 10.1113/jphysiol.1988.sp017239 PMC1190703

[27] Liu KS; Fetcho JR Laser ablations reveal functional relationships of segmental hindbrain neurons in zebrafish. Neuron 1999, 23, 325–335. 10399938 10.1016/s0896-6273(00)80783-7

[28] McLean DL; Fan J; Higashijima S; Hale ME; Fetcho JR A topographic map of recruitment in spinal cord. Nature 2007, 446, 71–75. 17330042 10.1038/nature05588

[29] McLean DL; Fetcho JR Spinal interneurons differentiate sequentially from those driving the fastest swimming movements in larval zebrafish to those driving the slowest ones. J. Neurosci 2009, 29, 13566–13577. 19864569 10.1523/JNEUROSCI.3277-09.2009 PMC2796107

[30] Forlano PM; Kim SD; Krzyminska ZM; Sisneros JA Catecholaminergic connectivity to the inner ear, central auditory, and vocal motor circuitry in the plainfin midshipman fish *Porichthys notatus*. J. Comp. Neurol 2014, 522, 2887–2927. 24715479 10.1002/cne.23596 PMC4107124

[31] Nagpal J; Herget U; Choi MK; Ryu S Anatomy, development, and plasticity of the neurosecretory hypothalamus in zebrafish. Cell Tissue Res 2019, 375, 5–22. 30109407 10.1007/s00441-018-2900-4

[32] Pagliaccio D; Luby JL; Bogdan R; Agrawal A; Gaffrey MS; Belden AC; Botteron KN; Harms MP; Barch DM Amygdala functional connectivity, HPA axis genetic variation, and life stress in children and relations to anxiety and emotion regulation. J. Abnorm. Psychol 2015, 124, 817–833. 26595470 10.1037/abn0000094 PMC4662045

[33] Porter BA; Mueller T The Zebrafish Amygdaloid Complex—Functional Ground Plan, Molecular Delineation, and Everted Topology. Front Neurosci 2020, 14, 608. 32765204 10.3389/fnins.2020.00608 PMC7378821

[34] Issa FA; Mazzochi C; Mock AF; Papazian DM Spinocerebellar ataxia type 13 mutant potassium channel alters neuronal excitability and causes locomotor deficits in zebrafish. J. Neurosci 2011, 31, 6831–6841. 21543613 10.1523/JNEUROSCI.6572-10.2011 PMC3101875

[35] Issa FA; Hall MK; Hatchett CJ; Weidner DA; Fiorenza AC; Schwalbe RA Compromised *N*-Glycosylation Processing of Kv3.1b Correlates with Perturbed Motor Neuron Structure and Locomotor Activity. Biology 2021, 10, 486. 34070741 10.3390/biology10060486 PMC8229559

[36] Issa FA; Mock AF; Sagasti A; Papazian DM Spinocerebellar ataxia type 13 mutation that is associated with disease onset in infancy disrupts axonal pathfinding during neuronal development. Dis. Models Mech 2012, 5, 921–929. 10.1242/dmm.010157 PMC3484873 22736459

