# Supplementary material for: Comparison of the Differing Impacts of Lowered N-Acetylglucosaminyltransferase-Ia/b Activity on Motor and Sensory Function in Zebrafish
Source: Int J Transl Med (Basel). Author manuscript; Available in PMC 2025 Oct 31. (PMC12573715; doi:10.3390/ijtm5030036)
Supplement: Supplementary Material [file NIHMS2116599-supplement-Supplementary_Material.pptx]

## Slide 1
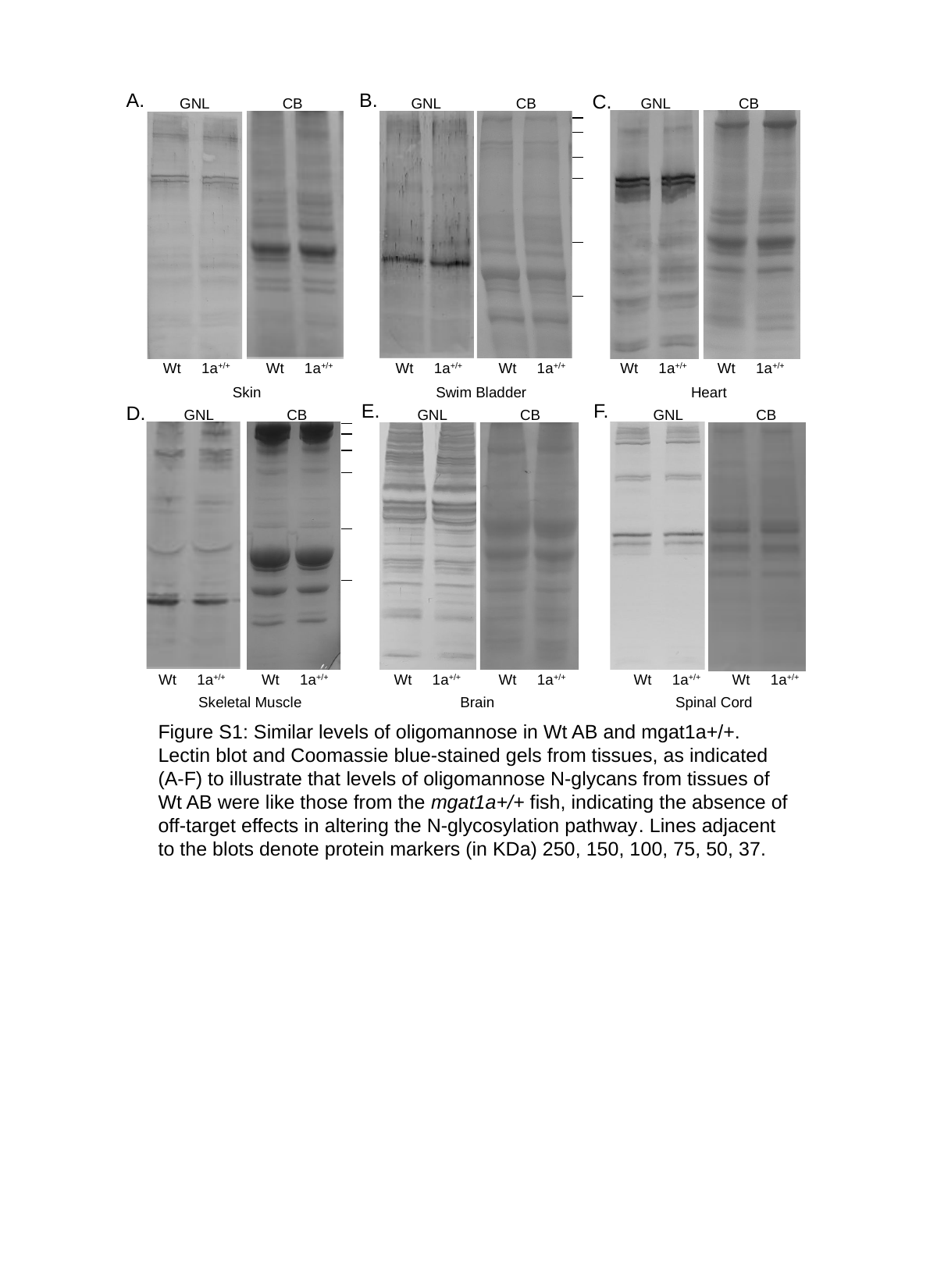

A.
B.
C.
GNL
CB
GNL
CB
GNL
CB
Wt 1a+/+
Wt 1a+/+
Wt 1a+/+
Wt 1a+/+
Wt 1a+/+
Wt 1a+/+
Skin
Swim Bladder
Heart
E.
F.
D.
GNL
CB
GNL
CB
GNL
CB
Wt 1a+/+
Wt 1a+/+
Wt 1a+/+
Wt 1a+/+
Wt 1a+/+
Wt 1a+/+
Skeletal Muscle
Brain
Spinal Cord
Figure S1: Similar levels of oligomannose in Wt AB and mgat1a+/+. Lectin blot and Coomassie blue-stained gels from tissues, as indicated (A-F) to illustrate that levels of oligomannose N-glycans from tissues of Wt AB were like those from the mgat1a+/+ fish, indicating the absence of off-target effects in altering the N-glycosylation pathway. Lines adjacent to the blots denote protein markers (in KDa) 250, 150, 100, 75, 50, 37.

## Slide 2
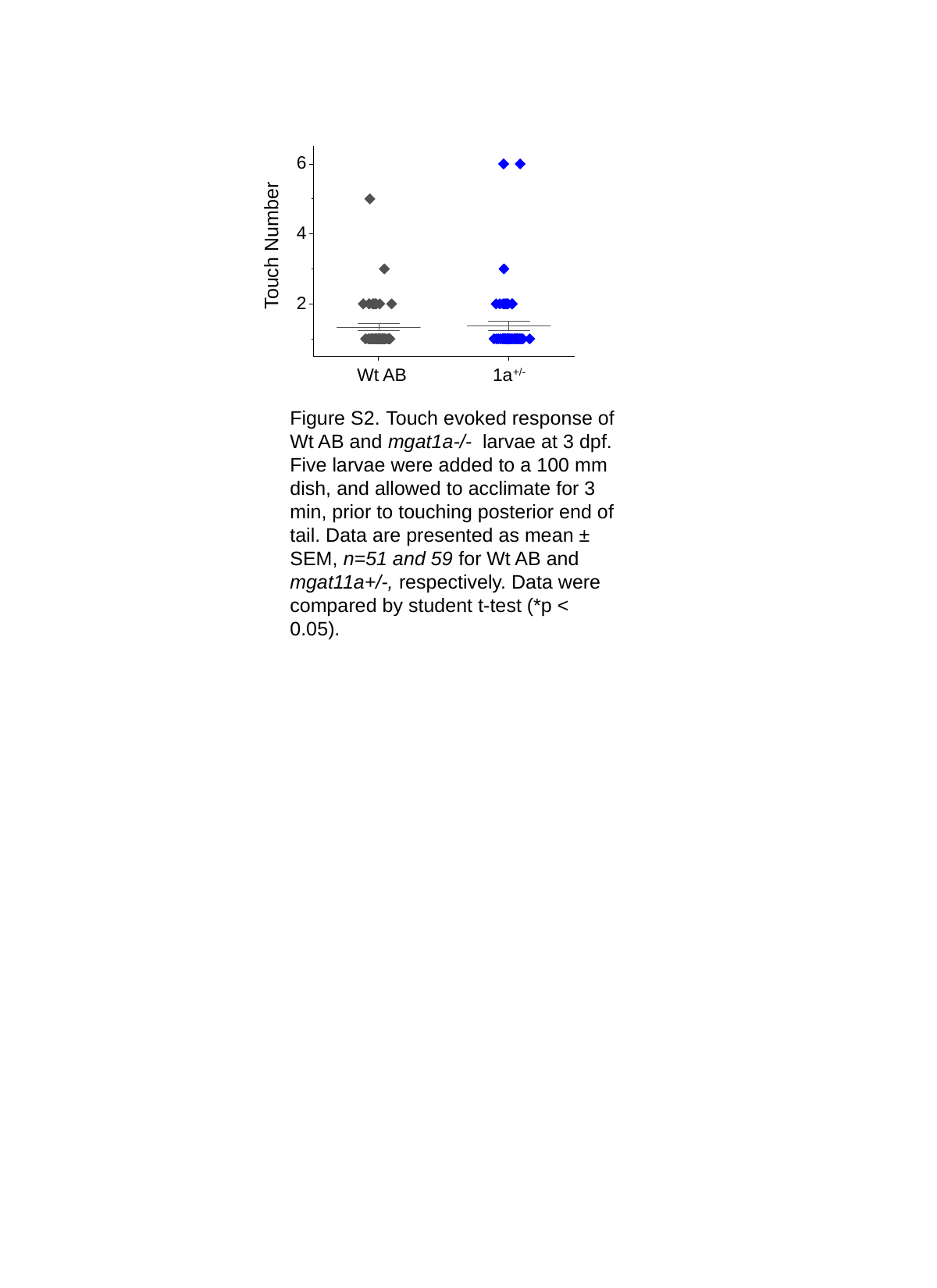

Touch Number
Wt AB 1a+/-
Figure S2. Touch evoked response of Wt AB and mgat1a-/- larvae at 3 dpf. Five larvae were added to a 100 mm dish, and allowed to acclimate for 3 min, prior to touching posterior end of tail. Data are presented as mean ± SEM, n=51 and 59 for Wt AB and mgat11a+/-, respectively. Data were compared by student t-test (*p < 0.05).
